# The Book World of Medicine and Science

**Published:** 1911-09-16

**Authors:** 


### The Book World of Medicine and Science.

### A MANUAL FOR THE SHIP'S DOCTOR.

Ship's Hygiene: Some New and Interesting Points. By W. Melville-Davidson, M.B., B.S. (Bristol: John Wright and Sons, Ltd., 1911. Pp. 87. Price 4s. net.)

Health on shipboard is undoubtedly claiming more and more attention as the years roll on, but whether the advances in hygiene are keeping pace with the increasing facilities of communication and the increasing number of passengers on steamships is a matter of some doubt. The completion of the Panama Canal will, it is feared by many, be followed by the dissemination of plague over a wider area from such a busy but infected centre as Hong-Kong, as well as by the introduction of yellow fever among the teeming populations of India and China.

The importance, both hygienically and commercially speaking, of precautionary measures, does not require to be emphasised, and owners of steamships are recognising that they have it in their power to do much to safeguard the health of passengers and crews, and by taking precautions in advance to minimise the vexatious delays of quarantine and to save expenses avoidable in many cases. Dr. Melville-Davidson has touched on only a few points in his book; but he has succeeded in compiling several highly interesting chapters, some of which contain the results of work on original and suggestive lines. After describing in detail the installation of water-filtering apparatus and the adaptation of mosquito screens to the exigencies of accommodation on shipboard, the author proceeds to relate his experiments with a bacillary virus against rats. This chapter is, indeed, the best in the book, and the story of the prolonged trial of the virus in some of the ships of the Booth Line is of great practical interest.

Commencing with a virus of ordinary strength and effecting therewith a considerable reduction in the number of rats on board, it was soon found that the rodents acquired an immunity and actually throve on the poison. Raising the virulence of the bacillus failed to produce corresponding results, and the laying down of the virus was temporarily discontinued. Then the interesting discovery was made that after a few months the number of rats began to decrease, and continued to decrease, although no virus was being exposed; the explanation being that the constant feeding of immune rats with a highly virulent virus had evidently produced in them a condition of "germ carrier," and thus the continued spread of the disease among the rats was rendered possible. The chapter itself must be read for the details of these experiments to be properly appreciated, for the above is but a bald outline, while the author's account is well worth reading.

The subject of cockroaches is one that Dr. Melville-Davidson has also studied, and he relates how he conducted some researches on the intestinal parasites of these creatures. He suggests that there is some connection between an amoeba found in the cockroach and the ætiology of beri-beri, while he attributes scurvy to a gregarina found also in the intestine of this insect. Without detracting in the least from the value of these suggestions, we could have wished that this chapter had been more systematically composed, and that further evidence from his own researches could have been imported into it.

In connection with the practical details of disinfection and the destruction of bugs, etc., the author has made good points, and the book, which is directed primarily to ship-owners, should more than justify its existence.

### A CONTRIBUTION TO EUGENICS.

The Treasury of Human Inheritance. Parts V. and VI., Section XIVa, Hæmophilia. By W. Bulloch, M.D., and Paul Fildes, M.B., B.C. 1911. (London: Dulau and Co.)

The Academic Aspect of the Science of National Eugenics. By Professor Karl Pearson.

At some golden future time—to be expected when the dream of royal philosophers has come true—we may see medical thought, led not by those who aim first at treating sick people or studying morbid tissues or maintaining sanitation, but by men more directed by, and blessed with better facility for gratifying, a thirst for the whole truth of their subject simply for that truth's own sake. This reflection is evoked by a searching study of the literature of the transmission of hæmophilia which has just appeared. The deliverances of distinguished clinicians on the subject of the inheritance of disease are, strangely enough, prone to include fragmentary information from a house-physician's notes, the view given in the leading British text-book, a little furbishing up of recollections of Weismann, a glance at Mendelism, a few platitudes coloured by prepossession in favour of, as the jingle goes, nurture as against nature—and nothing else at all. A foundation of personal work, or of knowledge derived from first-hand study of significant work by others (and on any clinico-pathological point one or other of these would be plainly evident) is wanting; yet opinions are advanced, to be duly quoted in the newspapers which do so abound now, there to be read by influential laymen. It is not the profession of this country alone which is to blame. At a recent congress on the Continent several European gentlemen famous for their skill with microscope or stethoscope enunciated dicta the insubstantiality or incorrectitude of which half an hour's looking into the matter would have demonstrated. The casual way in which these "bypaths of medicine" are pursued is shown up in Dr. Bulloch's notable résumé, a piece of critical work worth all the mediocre text-books (and they must positively run to thousands) published during the last ten years. A worker who corresponds with responsibly placed medical men throughout the civilised world, collects with their help 911 papers on his subject, and reads through almost every one of them—such an one, after all this effort, is not likely, as it were, to cry stinking fish without cause. And yet it is observed (p. 172): "It cannot be denied that without Grandivier's labours a large mass of literature would have been lost in oblivion. *While this, in our opinion, would have been desirable in a very large number of instances* . . . ." What finally pass the sieve are only 44 cases. The authors find from these that hæmophilia is a disease of males, that it is largely confined to the Teutonic race, that the transmission is solely through females, that "bleeder" families are large, with a proportion of male children greatly above the average. But quite definite conclusions have to be forgone pending the accumulation of a large number of well investigated cases. Accompanying the above is a little pamphlet by Professor Karl Pearson, entitled "The Academic Aspect of the Science of National Eugenics." It is a protest against the prejudging of various questions in the category of cause and effect, no matter how prepossessing the grounds may be for a preformed opinion. In effect it just repeats Huxley's warning against taking the "high priori" road. But whereas Huxley's arguments were open to any educated person's inspection and comprehension, the reasons given by Professor Pearson must necessarily be esoteric, depending as they do, finally, on the general applicability of the mathematical methods of measuring the degree of correlation of phenomena adopted by the biometric school. Thus, for instance, it is argued that no legislation in the interests of the offspring should be adopted as yet against employment of mothers, because from the statistics the Galton Eugenics Laboratory has investigated it appears that overcrowding and certain employments of the father have each twice as much relation to infantile mortality as has the mother's daily visit to the factory. Again, though the occupiers of back-to-back houses do die more frequently than others, yet it is claimed that since such people also receive lower wages, also drink more, and also crowd their rooms more, it becomes likely that they belong to a mentally and physically feebler class which drifts into the cheapest accommodation. Therefore legislation against back-to-back houses is illogical. But then, granting all this, both the above evils are feasible objects of legislation : drink, low wages, overcrowding, very difficult ones. Professor Pearson's claim for investigation is quite reasonable; but he must give full weight to practical considerations. The general tone of the earlier part of the pamphlet may, and most likely will, come to be justified, but this cannot be said of one or two particular statements. It is asserted that "Mendelism is having its day," and that this day will soon be yesterday. True, biometricians have produced cases of the transmission of certain attributes in a modified indefinite fashion very unlike the behaviour of a classical Mendelian unit, but to say at the present time that Mendelism is on its way to the limbo of exploded theories is uncommonly like an example of the "high priori" attitude which Professor Pearson condemns ably and incisively.

---

On Diseases of the Lungs and Pleuræ. By Sir R. Douglas Powell, Bart., and P. Horton-Smith Hartley, M.V.O., M.D., F.R.C.P. Fifth Edition. (London: H. K. Lewis, 136 Gower Street, W.C. 1911. Price 21s. net.)

The last edition of this text-book was published some fifteen years ago, and at the time won for itself a well-deserved reputation, since it dealt with diseases of the lungs in a broadly practical manner that appealed more especially to the general practitioner. During this decade and a half the diagnosis and treatment of pulmonary disease have made memorable advances. In the domain of pulmonary tuberculosis, for example, both the sanatorium and serum methods of treatment have been subjected to a strict investigation, and, although we are not yet in a position absolutely to dogmatise on these subjects, the results obtained have been of such a nature that broad generalisations such as ought to be found in text-books of this nature are possible. The authors are to be congratulated on the manner in which they have tackled the huge field of facts that awaited their recognition and discussion in view of these recent advances. They have brought their book up to date and have incorporated into it the soundest clinical teaching and the clearest enumeration of theory, so that it serves as an excellent text-book for the student who is preparing for his examinations or walking the wards. Much more important a matter is the careful attention that has been given to details of treatment. This is especially seen in the discussion with regard to the use of tuberculin. The authors have adopted the German system in which the dosage is stated in centimetres and fractions of a centimetre—a method which considerably simplifies the work of the prescriber. An interesting and very valuable feature of the book is the careful selection of illustrative cases. In most modern text-books such illustrations are sacrificed owing to exigencies of space—hence the marked difference in the practical value of such books when compared to the older manuals, of which the first edition of Fagge, for example, stands easily first. The value of such illustrative cases can scarcely be overestimated; in this book they have been so well selected that everyone is instructive. Excellent illustrations, really elucidative of the text, add to the value of the work, which is one deserving of a permanent place on the library shelves of the practitioner.

On Acute Intestinal Toxæmia in Infants. By Ralph Vincent, M.D. (London: Baillière, Tindall and Cox. 1911. Pp. 83. Price 3s. 6d.)

Dr. Ralph Vincent has recently issued in book form an address which he delivered before the Glasgow Obstetrical and Gynæcological Society on an experimental investigation of the ætiology and pathology of epidemic or summer diarrhœa. In the book we find elaborated his well-known views on the dangers of feeding infants on milk which has been boiled. The author is at great pains to substantiate his argument, and the major portion of the book is taken up with a long and detailed account of certain experiments on forty unfortunate kittens, who were fed on milk inoculated with various bacteria. The lengthy details make somewhat tedious reading, and no very characteristic or definite post-mortem findings are recorded, in spite of thorough microscopical investigations. The author holds a brief for the beneficent action of the lactic acid producing bacteria, but his case is rather encumbered by long quotations and personal verbosity. But his earnestness commands some attention, although his conclusions may not meet with universal acceptance. Dr. Vincent thinks that the boiling of milk is "a much more effective prescription for the production of tuberculosis than the administration of milk containing, now and again, a few tubercle bacilli." He is perhaps doing good work in inveighing against the indiscriminate routine boiling of milk for infants' food, and his large experience of infants fed on raw milk is not lightly to be disregarded. The book contains a large number of excellent plates which will be of great interest to students of elementary bacteriology.

Crime and Insanity. By Charles Mercier, M.D., F.R.C.P. The Home University Library. (London: Williams and Norgate. 1911. Price 1s.)

If the editors of the Home University Library have distinguished themselves by the number of excellent text-books they have brought out in this series, we can say none the less truly that Dr. Charles Mercier has also distinguished himself in his contribution to the library. From his pen we have been accustomed to expect not only valuable knowledge but also originality of conception permeated by a characteristic vein of humour. This little volume, on a subject hardly to be considered popular, well vindicates its inclusion in the instructive series now being so successfully published. The charm of Dr. Mercier's style is sustained throughout his discourse, and this must surely be counted a factor in the growth of the favour accorded to his views on insanity. To quote from a short treatise so easily obtainable is scarcely necessary, so we content ourselves with affirming that the subject of crime is treated in so interesting and logical a fashion that the reader quickly attains that state so frequently coveted of being simultaneously instructed and entertained.

